# 4-Chloro-*N*′-(2-methoxy­benzyl­idene)benzohydrazide

**DOI:** 10.1107/S1600536809010186

**Published:** 2009-03-25

**Authors:** Hong-Yuan Wu

**Affiliations:** a College of Chemistry and Chemical Engineering, Qiqihar University, Qiqihar 161006, People’s Republic of China

## Abstract

The title compound, C_15_H_13_ClN_2_O_2_, was prepared by the reaction of 3-methoxy­benzaldehyde and 4-chloro­benzo­hydrazide in methanol. The asymmetric unit consists of two unique molecules, which are linked together in the form of a cross by N—H⋯O and N—H⋯N hydrogen bonds. The dihedral angles between the two benzene rings in the mol­ecules are 77.3 (1) and 44.1 (1)°. In the crystal structure, mol­ecules are linked through inter­molecular N—H⋯O hydrogen bonds, forming chains along the *a* axis.

## Related literature

For the crystal structures of hydrazone derivatives, see: Singh *et al.* (2007 ▶); Fun *et al.* (2008 ▶); Khaledi *et al.* (2008 ▶); Alhadi *et al.* (2008 ▶). For bond-length data, see: Allen *et al.* (1987 ▶).
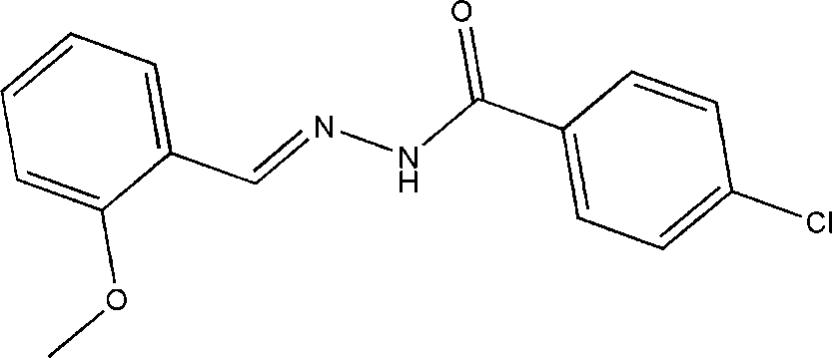


## Experimental

### 

#### Crystal data

C_15_H_13_ClN_2_O_2_
*M* *_r_* = 288.72
Triclinic, 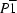

*a* = 7.802 (2) Å
*b* = 13.395 (3) Å
*c* = 14.599 (2) Å
α = 93.298 (2)°
β = 100.945 (3)°
γ = 106.055 (2)°
*V* = 1429.7 (5) Å^3^
*Z* = 4
Mo *K*α radiation
μ = 0.27 mm^−1^
*T* = 298 K
0.13 × 0.13 × 0.12 mm

#### Data collection

Bruker APEXII CCD area-detector diffractometer
Absorption correction: multi-scan (*SADABS*; Sheldrick, 2004 ▶) *T*_min_ = 0.963, *T*_max_ = 0.967
8597 measured reflections
6134 independent reflections
3465 reflections with *I* > 2σ(*I*)
*R*_int_ = 0.018

#### Refinement

*R*[*F* ^2^ > 2σ(*F* ^2^)] = 0.054
*wR*(*F* ^2^) = 0.153
*S* = 1.03
6134 reflections
363 parameters
H-atom parameters constrained
Δρ_max_ = 0.27 e Å^−3^
Δρ_min_ = −0.37 e Å^−3^

### 

Data collection: *APEX2* (Bruker, 2004 ▶); cell refinement: *SAINT* (Bruker, 2004 ▶); data reduction: *SAINT*; program(s) used to solve structure: *SHELXS97* (Sheldrick, 2008 ▶); program(s) used to refine structure: *SHELXL97* (Sheldrick, 2008 ▶); molecular graphics: *ORTEPIII* (Burnett & Johnson, 1996 ▶), *ORTEP-3 for Windows* (Farrugia, 1997 ▶) and *PLATON* (Spek, 2009 ▶); software used to prepare material for publication: *SHELXL97*.

## Supplementary Material

Crystal structure: contains datablocks global, I. DOI: 10.1107/S1600536809010186/dn2433sup1.cif

Structure factors: contains datablocks I. DOI: 10.1107/S1600536809010186/dn2433Isup2.hkl

Additional supplementary materials:  crystallographic information; 3D view; checkCIF report

## Figures and Tables

**Table 1:** Hydrogen-bond geometry (Å, °)

*D*—H⋯*A*	*D*—H	H⋯*A*	*D*⋯*A*	*D*—H⋯*A*
N1—H1⋯O3^i^	0.86	2.01	2.840 (3)	162
N3—H3⋯O1	0.86	2.14	2.897 (3)	147
N3—H3⋯N2	0.86	2.57	3.292 (3)	142

Symmetry code: (i) 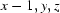.

**Fig. 1.**
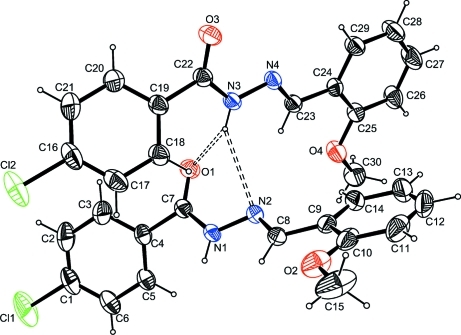
Molecular structure of the title compound with the atom-labeling scheme. Ellipsoids are drawn at the 30% probability level. H atoms are represented as small spheres of arbitrary radii. H bonds are shown as dashed lines.

**Fig. 2.**
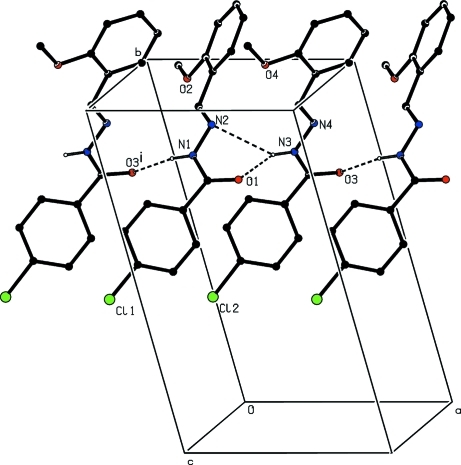
Partial packing view showing the chain formed by N-H···O hydrogen bonds shown as dashed lines. H atoms not involved in hydrogen bonding have been omitted for clarity. [Symmetry code: (i) x-1, y, z]

## supplementary crystallographic information

### Comment

Recently, the crystal structures of hydrazone derivatives have been widely reported (Singh *et al.*, 2007; Fun *et al.*, 2008; Khaledi *et al.*, 2008; Alhadi *et al.*, 2008). As an ongoing study of such compounds, the title new compound was reported here.

The asymmetric unit of the title compound consists of two crossed molecules, which are linked together by intramolecular N–H···O and N–H···N hydrogen bonds (Fig. 1 and Table 1). The dihedral angles between the two benzene rings in the molecules are 77.3 (1) and 44.1 (1)°, respectively. All the bond lengths are within normal ranges (Allen *et al.*, 1987).

In the crystal structure, molecules are linked through intermolecular N–H···O hydrogen bonds (Table 1), forming chains along the *a* axis (Fig. 2).

### Experimental

2-Methoxybenzaldehyde (1.0 mmol) and 4-chlorobenzohydrazide (1.0 mmol) were dissolved in a methanol solution. The mixture was stirred at room temperature for 10 min to give a clear colorless solution. The solution was left to slow evaporate for a few days, yielding colorless needle-shaped crystals.

### Refinement

All H atoms attached to C atoms and N atom were fixed geometrically and treated as riding with C—H = 0.96 Å (methyl) or 0.93 Å (aromatic) and N—H = 0.86 Å with Uĩso(H) = xUeq(C or N) with x=1.2 or 1.5 for methyl group.

### Crystal data

**Table:**

C_15_H_13_ClN_2_O_2_	*Z* = 4
*M**_r_* = 288.72	*F*(000) = 600
Triclinic, *P*1	*D*_x_ = 1.341 Mg m^−^^3^
Hall symbol: -P 1	Mo *K*α radiation, λ = 0.71073 Å
*a* = 7.802 (2) Å	Cell parameters from 1723 reflections
*b* = 13.395 (3) Å	θ = 2.5–24.5°
*c* = 14.599 (2) Å	µ = 0.27 mm^−^^1^
α = 93.298 (2)°	*T* = 298 K
β = 100.945 (3)°	Cut from needle, colorless
γ = 106.055 (2)°	0.13 × 0.13 × 0.12 mm
*V* = 1429.7 (5) Å^3^	

### Data collection

**Table:**

Bruker APEXII CCD area-detector diffractometer	6134 independent reflections
Radiation source: fine-focus sealed tube	3465 reflections with *I* > 2σ(*I*)
graphite	*R*_int_ = 0.018
ω scans	θ_max_ = 27.0°, θ_min_ = 1.4°
Absorption correction: multi-scan (*SADABS*; Sheldrick, 2004)	*h* = −9→9
*T*_min_ = 0.963, *T*_max_ = 0.967	*k* = −16→17
8597 measured reflections	*l* = −18→14

### Refinement

**Table:**

Refinement on *F*^2^	Primary atom site location: structure-invariant direct methods
Least-squares matrix: full	Secondary atom site location: difference Fourier map
*R*[*F*^2^ > 2σ(*F*^2^)] = 0.054	Hydrogen site location: inferred from neighbouring sites
*wR*(*F*^2^) = 0.153	H-atom parameters constrained
*S* = 1.03	*w* = 1/[σ^2^(*F*_o_^2^) + (0.0595*P*)^2^ + 0.2838*P*] where *P* = (*F*_o_^2^ + 2*F*_c_^2^)/3
6134 reflections	(Δ/σ)_max_ = 0.001
363 parameters	Δρ_max_ = 0.27 e Å^−^^3^
0 restraints	Δρ_min_ = −0.37 e Å^−^^3^

### Special details

**Table:**

Geometry. All esds (except the esd in the dihedral angle between two l.s. planes) are estimated using the full covariance matrix. The cell esds are taken into account individually in the estimation of esds in distances, angles and torsion angles; correlations between esds in cell parameters are only used when they are defined by crystal symmetry. An approximate (isotropic) treatment of cell esds is used for estimating esds involving l.s. planes.
Refinement. Refinement of *F*^2^ against ALL reflections. The weighted *R*-factor *wR* and goodness of fit *S* are based on *F*^2^, conventional *R*-factors *R* are based on *F*, with *F* set to zero for negative *F*^2^. The threshold expression of *F*^2^ > σ(*F*^2^) is used only for calculating *R*-factors(gt) *etc*. and is not relevant to the choice of reflections for refinement. *R*-factors based on *F*^2^ are statistically about twice as large as those based on *F*, and *R*- factors based on ALL data will be even larger.

### Fractional atomic coordinates and isotropic or equivalent isotropic displacement parameters (Å^2^)

**Table:**

	*x*	*y*	*z*	*U*_iso_*/*U*_eq_	
Cl1	−0.36428 (18)	0.35966 (9)	0.40462 (10)	0.1543 (6)	
Cl2	0.01821 (14)	0.31778 (8)	0.07426 (8)	0.1277 (4)	
N1	0.1823 (3)	0.75724 (14)	0.25213 (13)	0.0483 (5)	
H1	0.0681	0.7446	0.2274	0.058*	
N2	0.3077 (3)	0.84442 (15)	0.23333 (14)	0.0507 (5)	
N3	0.6661 (2)	0.76354 (14)	0.22293 (14)	0.0500 (5)	
H3	0.5645	0.7571	0.2402	0.060*	
N4	0.8123 (2)	0.85210 (14)	0.25581 (13)	0.0475 (5)	
O1	0.3995 (2)	0.69948 (13)	0.33805 (12)	0.0609 (5)	
O2	0.1670 (4)	0.9429 (2)	−0.00901 (17)	0.0918 (7)	
O3	0.8240 (2)	0.69020 (14)	0.13798 (12)	0.0633 (5)	
O4	0.7265 (3)	1.05068 (14)	0.44551 (13)	0.0713 (5)	
C1	−0.1898 (5)	0.4568 (3)	0.3777 (2)	0.0879 (10)	
C2	−0.0336 (6)	0.4345 (2)	0.3656 (3)	0.0975 (11)	
H2	−0.0226	0.3678	0.3719	0.117*	
C3	0.1072 (4)	0.5114 (2)	0.3440 (2)	0.0776 (8)	
H3A	0.2144	0.4971	0.3372	0.093*	
C4	0.0877 (3)	0.60967 (18)	0.33272 (17)	0.0528 (6)	
C5	−0.0703 (3)	0.6296 (2)	0.34588 (17)	0.0581 (6)	
H5	−0.0835	0.6958	0.3389	0.070*	
C6	−0.2094 (4)	0.5536 (2)	0.3691 (2)	0.0732 (8)	
H6	−0.3147	0.5683	0.3787	0.088*	
C7	0.2386 (3)	0.69232 (18)	0.30901 (17)	0.0493 (6)	
C8	0.2455 (3)	0.88706 (18)	0.16387 (18)	0.0525 (6)	
H8	0.1296	0.8545	0.1273	0.063*	
C9	0.3533 (4)	0.9858 (2)	0.1410 (2)	0.0621 (7)	
C10	0.3066 (5)	1.0144 (3)	0.0507 (3)	0.0809 (10)	
C11	0.4045 (7)	1.1090 (3)	0.0286 (4)	0.1179 (17)	
H11	0.3749	1.1280	−0.0313	0.141*	
C12	0.5443 (7)	1.1749 (3)	0.0941 (5)	0.136 (2)	
H12	0.6091	1.2385	0.0783	0.164*	
C13	0.5913 (5)	1.1490 (3)	0.1832 (4)	0.1147 (16)	
H13	0.6873	1.1945	0.2272	0.138*	
C14	0.4943 (4)	1.0544 (2)	0.2068 (3)	0.0788 (9)	
H14	0.5240	1.0368	0.2673	0.095*	
C15	0.1087 (6)	0.9678 (4)	−0.1012 (3)	0.1261 (17)	
H15A	0.0833	1.0339	−0.0971	0.189*	
H15B	0.0002	0.9144	−0.1330	0.189*	
H15C	0.2033	0.9720	−0.1356	0.189*	
C16	0.2096 (4)	0.4261 (2)	0.0957 (2)	0.0792 (9)	
C17	0.1896 (4)	0.5239 (2)	0.0987 (2)	0.0723 (8)	
H17	0.0737	0.5328	0.0875	0.087*	
C18	0.3427 (3)	0.6096 (2)	0.11840 (17)	0.0590 (7)	
H18	0.3294	0.6765	0.1203	0.071*	
C19	0.5153 (3)	0.59732 (19)	0.13536 (17)	0.0528 (6)	
C20	0.5314 (4)	0.4971 (2)	0.1296 (2)	0.0737 (8)	
H20	0.6466	0.4872	0.1391	0.088*	
C21	0.3781 (5)	0.4117 (2)	0.1100 (2)	0.0872 (10)	
H21	0.3898	0.3444	0.1066	0.105*	
C22	0.6829 (3)	0.68759 (19)	0.16411 (17)	0.0507 (6)	
C23	0.7862 (3)	0.91383 (17)	0.31774 (16)	0.0469 (5)	
H23	0.6764	0.8963	0.3378	0.056*	
C24	0.9252 (3)	1.01116 (17)	0.35755 (16)	0.0459 (5)	
C25	0.8937 (3)	1.08002 (18)	0.42280 (17)	0.0522 (6)	
C26	1.0264 (4)	1.1724 (2)	0.4600 (2)	0.0720 (8)	
H26	1.0043	1.2182	0.5036	0.086*	
C27	1.1899 (4)	1.1971 (2)	0.4332 (2)	0.0871 (10)	
H27	1.2791	1.2593	0.4592	0.105*	
C28	1.2236 (4)	1.1312 (2)	0.3686 (2)	0.0836 (9)	
H28	1.3348	1.1485	0.3501	0.100*	
C29	1.0921 (3)	1.0396 (2)	0.33148 (19)	0.0620 (7)	
H29	1.1154	0.9951	0.2873	0.074*	
C30	0.6940 (5)	1.1163 (2)	0.5169 (2)	0.0871 (10)	
H30A	0.7792	1.1197	0.5747	0.131*	
H30B	0.5717	1.0880	0.5256	0.131*	
H30C	0.7093	1.1853	0.4983	0.131*	

### Atomic displacement parameters (Å^2^)

**Table:**

	*U*^11^	*U*^22^	*U*^33^	*U*^12^	*U*^13^	*U*^23^
Cl1	0.1353 (10)	0.1066 (8)	0.1913 (13)	−0.0377 (7)	0.0607 (9)	0.0557 (8)
Cl2	0.0964 (7)	0.0919 (7)	0.1465 (9)	−0.0403 (5)	0.0170 (7)	−0.0068 (6)
N1	0.0392 (10)	0.0469 (11)	0.0584 (12)	0.0070 (8)	0.0157 (9)	0.0148 (9)
N2	0.0449 (11)	0.0445 (11)	0.0650 (13)	0.0100 (9)	0.0201 (10)	0.0137 (10)
N3	0.0369 (10)	0.0447 (11)	0.0629 (12)	0.0044 (8)	0.0123 (9)	−0.0067 (9)
N4	0.0416 (10)	0.0417 (10)	0.0531 (12)	0.0038 (8)	0.0099 (9)	−0.0017 (9)
O1	0.0496 (11)	0.0653 (11)	0.0683 (11)	0.0146 (9)	0.0135 (9)	0.0200 (9)
O2	0.1113 (19)	0.1159 (19)	0.0810 (15)	0.0610 (17)	0.0465 (14)	0.0513 (14)
O3	0.0462 (10)	0.0679 (11)	0.0683 (11)	0.0056 (8)	0.0174 (9)	−0.0139 (9)
O4	0.0701 (12)	0.0601 (11)	0.0799 (13)	0.0030 (9)	0.0377 (10)	−0.0136 (9)
C1	0.084 (2)	0.069 (2)	0.089 (2)	−0.0181 (17)	0.0222 (19)	0.0211 (17)
C2	0.120 (3)	0.0505 (18)	0.116 (3)	0.0063 (19)	0.031 (2)	0.0317 (18)
C3	0.085 (2)	0.0574 (18)	0.093 (2)	0.0173 (16)	0.0265 (18)	0.0241 (16)
C4	0.0568 (15)	0.0484 (14)	0.0494 (14)	0.0067 (11)	0.0136 (12)	0.0114 (11)
C5	0.0577 (16)	0.0557 (15)	0.0558 (15)	0.0034 (12)	0.0184 (13)	0.0099 (12)
C6	0.0646 (18)	0.074 (2)	0.0690 (18)	−0.0059 (15)	0.0227 (15)	0.0112 (15)
C7	0.0482 (14)	0.0482 (14)	0.0504 (14)	0.0092 (11)	0.0149 (11)	0.0060 (11)
C8	0.0474 (14)	0.0504 (14)	0.0656 (16)	0.0153 (11)	0.0237 (12)	0.0118 (12)
C9	0.0584 (16)	0.0539 (15)	0.095 (2)	0.0273 (13)	0.0452 (16)	0.0300 (15)
C10	0.090 (2)	0.074 (2)	0.121 (3)	0.0507 (19)	0.073 (2)	0.050 (2)
C11	0.133 (4)	0.096 (3)	0.192 (5)	0.070 (3)	0.119 (4)	0.090 (3)
C12	0.130 (4)	0.064 (3)	0.271 (7)	0.046 (3)	0.136 (5)	0.077 (4)
C13	0.086 (3)	0.054 (2)	0.220 (5)	0.0158 (18)	0.077 (3)	0.019 (3)
C14	0.0608 (18)	0.0509 (16)	0.134 (3)	0.0156 (14)	0.0435 (19)	0.0099 (17)
C15	0.156 (4)	0.202 (5)	0.092 (3)	0.127 (4)	0.068 (3)	0.083 (3)
C16	0.0652 (19)	0.065 (2)	0.082 (2)	−0.0149 (15)	0.0114 (16)	−0.0119 (15)
C17	0.0485 (16)	0.080 (2)	0.0712 (18)	0.0001 (14)	0.0050 (13)	−0.0130 (15)
C18	0.0504 (15)	0.0569 (15)	0.0592 (16)	0.0073 (12)	0.0039 (12)	−0.0108 (12)
C19	0.0448 (14)	0.0523 (14)	0.0529 (14)	0.0057 (11)	0.0074 (11)	−0.0083 (11)
C20	0.0582 (17)	0.0572 (17)	0.096 (2)	0.0125 (14)	0.0054 (16)	−0.0127 (15)
C21	0.085 (2)	0.0491 (17)	0.111 (3)	0.0045 (16)	0.010 (2)	−0.0108 (16)
C22	0.0442 (14)	0.0513 (14)	0.0520 (14)	0.0100 (11)	0.0075 (11)	−0.0027 (11)
C23	0.0418 (13)	0.0452 (13)	0.0508 (14)	0.0087 (10)	0.0099 (11)	0.0019 (11)
C24	0.0462 (13)	0.0399 (12)	0.0468 (13)	0.0064 (10)	0.0086 (10)	0.0009 (10)
C25	0.0543 (15)	0.0483 (14)	0.0512 (14)	0.0078 (11)	0.0158 (12)	0.0028 (11)
C26	0.078 (2)	0.0516 (16)	0.0759 (19)	0.0005 (14)	0.0237 (16)	−0.0122 (14)
C27	0.068 (2)	0.0605 (18)	0.108 (3)	−0.0174 (15)	0.0229 (18)	−0.0240 (17)
C28	0.0556 (17)	0.073 (2)	0.107 (2)	−0.0082 (14)	0.0319 (17)	−0.0170 (18)
C29	0.0504 (15)	0.0571 (16)	0.0721 (17)	0.0047 (12)	0.0194 (13)	−0.0091 (13)
C30	0.101 (2)	0.072 (2)	0.095 (2)	0.0188 (18)	0.055 (2)	−0.0112 (17)

### Geometric parameters (Å, °)

**Table:**

Cl1—C1	1.727 (3)	C12—C13	1.374 (7)
Cl2—C16	1.733 (3)	C12—H12	0.9300
N1—C7	1.340 (3)	C13—C14	1.384 (5)
N1—N2	1.379 (2)	C13—H13	0.9300
N1—H1	0.8600	C14—H14	0.9300
N2—C8	1.274 (3)	C15—H15A	0.9600
N3—C22	1.344 (3)	C15—H15B	0.9600
N3—N4	1.384 (2)	C15—H15C	0.9600
N3—H3	0.8600	C16—C21	1.360 (4)
N4—C23	1.273 (3)	C16—C17	1.361 (4)
O1—C7	1.222 (3)	C17—C18	1.379 (4)
O2—C10	1.353 (4)	C17—H17	0.9300
O2—C15	1.426 (4)	C18—C19	1.379 (3)
O3—C22	1.224 (3)	C18—H18	0.9300
O4—C25	1.364 (3)	C19—C20	1.382 (4)
O4—C30	1.429 (3)	C19—C22	1.485 (3)
C1—C6	1.356 (5)	C20—C21	1.377 (4)
C1—C2	1.372 (5)	C20—H20	0.9300
C2—C3	1.382 (4)	C21—H21	0.9300
C2—H2	0.9300	C23—C24	1.451 (3)
C3—C4	1.382 (4)	C23—H23	0.9300
C3—H3A	0.9300	C24—C29	1.384 (3)
C4—C5	1.377 (3)	C24—C25	1.390 (3)
C4—C7	1.486 (3)	C25—C26	1.378 (3)
C5—C6	1.377 (3)	C26—C27	1.364 (4)
C5—H5	0.9300	C26—H26	0.9300
C6—H6	0.9300	C27—C28	1.365 (4)
C8—C9	1.453 (3)	C27—H27	0.9300
C8—H8	0.9300	C28—C29	1.367 (4)
C9—C14	1.385 (4)	C28—H28	0.9300
C9—C10	1.402 (4)	C29—H29	0.9300
C10—C11	1.378 (5)	C30—H30A	0.9600
C11—C12	1.363 (7)	C30—H30B	0.9600
C11—H11	0.9300	C30—H30C	0.9600
			
C7—N1—N2	119.90 (19)	O2—C15—H15B	109.5
C7—N1—H1	120.0	H15A—C15—H15B	109.5
N2—N1—H1	120.0	O2—C15—H15C	109.5
C8—N2—N1	113.9 (2)	H15A—C15—H15C	109.5
C22—N3—N4	120.04 (19)	H15B—C15—H15C	109.5
C22—N3—H3	120.0	C21—C16—C17	121.0 (3)
N4—N3—H3	120.0	C21—C16—Cl2	119.2 (3)
C23—N4—N3	114.35 (19)	C17—C16—Cl2	119.8 (3)
C10—O2—C15	118.5 (3)	C16—C17—C18	119.4 (3)
C25—O4—C30	117.5 (2)	C16—C17—H17	120.3
C6—C1—C2	121.2 (3)	C18—C17—H17	120.3
C6—C1—Cl1	119.6 (3)	C17—C18—C19	120.8 (3)
C2—C1—Cl1	119.2 (3)	C17—C18—H18	119.6
C1—C2—C3	119.9 (3)	C19—C18—H18	119.6
C1—C2—H2	120.0	C18—C19—C20	118.5 (2)
C3—C2—H2	120.0	C18—C19—C22	122.2 (2)
C2—C3—C4	119.6 (3)	C20—C19—C22	119.2 (2)
C2—C3—H3A	120.2	C21—C20—C19	120.5 (3)
C4—C3—H3A	120.2	C21—C20—H20	119.8
C5—C4—C3	119.0 (2)	C19—C20—H20	119.8
C5—C4—C7	121.3 (2)	C16—C21—C20	119.8 (3)
C3—C4—C7	119.7 (2)	C16—C21—H21	120.1
C4—C5—C6	121.4 (3)	C20—C21—H21	120.1
C4—C5—H5	119.3	O3—C22—N3	123.7 (2)
C6—C5—H5	119.3	O3—C22—C19	122.4 (2)
C1—C6—C5	118.8 (3)	N3—C22—C19	113.9 (2)
C1—C6—H6	120.6	N4—C23—C24	120.9 (2)
C5—C6—H6	120.6	N4—C23—H23	119.5
O1—C7—N1	123.1 (2)	C24—C23—H23	119.5
O1—C7—C4	122.8 (2)	C29—C24—C25	117.6 (2)
N1—C7—C4	114.1 (2)	C29—C24—C23	121.7 (2)
N2—C8—C9	120.9 (2)	C25—C24—C23	120.7 (2)
N2—C8—H8	119.5	O4—C25—C26	123.6 (2)
C9—C8—H8	119.5	O4—C25—C24	116.1 (2)
C14—C9—C10	119.3 (3)	C26—C25—C24	120.3 (2)
C14—C9—C8	121.9 (3)	C27—C26—C25	120.3 (3)
C10—C9—C8	118.7 (3)	C27—C26—H26	119.8
O2—C10—C11	125.2 (4)	C25—C26—H26	119.8
O2—C10—C9	115.4 (3)	C26—C27—C28	120.5 (3)
C11—C10—C9	119.4 (4)	C26—C27—H27	119.7
C12—C11—C10	120.4 (5)	C28—C27—H27	119.7
C12—C11—H11	119.8	C27—C28—C29	119.3 (3)
C10—C11—H11	119.8	C27—C28—H28	120.4
C11—C12—C13	121.2 (4)	C29—C28—H28	120.4
C11—C12—H12	119.4	C28—C29—C24	122.0 (2)
C13—C12—H12	119.4	C28—C29—H29	119.0
C12—C13—C14	119.3 (4)	C24—C29—H29	119.0
C12—C13—H13	120.3	O4—C30—H30A	109.5
C14—C13—H13	120.3	O4—C30—H30B	109.5
C13—C14—C9	120.4 (4)	H30A—C30—H30B	109.5
C13—C14—H14	119.8	O4—C30—H30C	109.5
C9—C14—H14	119.8	H30A—C30—H30C	109.5
O2—C15—H15A	109.5	H30B—C30—H30C	109.5

### Hydrogen-bond geometry (Å, °)

**Table:**

*D*—H···*A*	*D*—H	H···*A*	*D*···*A*	*D*—H···*A*
N1—H1···O3^i^	0.86	2.01	2.840 (3)	162
N3—H3···O1	0.86	2.14	2.897 (3)	147
N3—H3···N2	0.86	2.57	3.292 (3)	142

Symmetry codes: (i) *x*−1, *y*, *z*.
